# Assessment of Skin-Brain Interactions in Atopic Dermatitis Using Wearable EEG: An Exploratory Clinical Investigation

**DOI:** 10.7759/cureus.102120

**Published:** 2026-01-22

**Authors:** Maheshvari N Patel, Nayan Patel, Apeksha Merja

**Affiliations:** 1 Clinical Research, NovoBliss Research Private Limited, Ahmedabad, IND; 2 Pharmacology, Swaminarayan University, Ahmedabad, IND; 3 Clinical Research Operations, NovoBliss Research Private Limited, Ahmedabad, IND; 4 Sub-Investigator, NovoBliss Research Private Limited, Ahmedabad, IND

**Keywords:** atopic dermatitis, electroencephalography (eeg), exploratory validation study, neurosensory evaluation, skin–brain interaction, wearable eeg technology

## Abstract

Background

Atopic dermatitis (AD) is associated with heightened neurosensory perception, including itch and discomfort, which are traditionally assessed using subjective or clinical measures. Objective evaluation of central nervous system involvement in AD remains limited. Wearable electroencephalography (EEG) devices offer a non-invasive approach to capture neurosensory responses and may serve as objective tools for assessing skin-brain interactions in neurosensorial dermatology.

Objective

This exploratory clinical investigation aimed to validate the feasibility, reliability, and sensitivity of a wearable EEG headband for neurosensory evaluation in adults with mild to moderate AD following topical application of a marketed product compared with control (water). Correlation with dermatological evaluations was included as supportive evidence to contextualize EEG findings.

Methodology

This randomized, double-blind, control-arm, validation study enrolled adults aged 18-65 years with mild to moderate AD. Participants were randomized in a 1:1 ratio to receive either a marketed topical product or control (water). EEG recordings were obtained at baseline, following standardized 10% v/v lactic acid stimulation, and after product or control application during a single study visit. Changes in alpha (α), beta (β), delta (δ), and theta (θ) brainwave activity were analyzed to assess neurosensory responses. Dermatological evaluations were performed concurrently to support interpretation of EEG outcomes.

Results

EEG recordings demonstrated stable neurosensory patterns at baseline, followed by marked increases in cortical activity across multiple frequency bands after 10% v/v lactic acid stimulation, indicating heightened sensory processing. Following test product application, EEG profiles returned toward a calm and regulated state, with reduced low- and mid-frequency activity and stabilization of α rhythms, suggesting neurosensory recovery and tolerability. In contrast, control application did not result in EEG normalization, with sustained or increased activity across frequency bands, indicating persistent sensory reactivity. Dermatological assessments followed the same directional trends as EEG findings, supporting the physiological relevance of the neurosensory data.

Conclusion

This study validates wearable EEG as a feasible and sensitive methodology for objective neurosensory assessment in AD. EEG successfully differentiated between irritation-induced activation, product-related soothing, and control response, with supportive alignment from dermatological evaluations. These findings support the integration of EEG-based neurosensory endpoints in future dermatological research and early-phase product evaluation.

## Introduction

Atopic dermatitis (AD) is a chronic inflammatory skin disease characterized by epidermal barrier dysfunction, pruritus, and heightened neurosensory responsiveness, which significantly affect quality of life. Traditional clinical assessments rely heavily on visible signs and patient-reported outcomes, which may not fully capture underlying neurophysiological processes contributing to sensory perception and itch. This highlights the need for objective tools capable of evaluating central nervous system (CNS) involvement in AD [1-3].

Electroencephalography (EEG) is a non-invasive neurophysiological technique with high temporal resolution that measures cortical activity underlying sensory, cognitive, and affective processing. EEG frequency bands, such as alpha (α), beta (β), and theta (θ), are well-established markers linked to relaxation, alertness, drowsiness, sensory modulation, and itch perception [4-7]. Advances in wearable EEG systems, including dry-electrode devices, have made it feasible to conduct reliable neurosensory measurements in clinical settings with minimal setup burden [8,9].

EEG has been increasingly explored in dermatology to characterize neural correlates of itch and sensory discomfort. Studies in AD subjects demonstrate altered oscillatory activity, particularly in the α band, during itch induction or exacerbation, supporting the potential of EEG as an objective indicator of neurosensory activity [10-12]. Wearable devices such as the EEG headband have shown acceptable signal quality for clinical research applications, capturing reproducible neural patterns across multiple frequency bands [13,14]. 

This exploratory study aims to validate the feasibility and reliability of a wearable EEG headband for neurosensory assessment in adults with mild to moderate AD following topical product application. The primary focus is to determine whether EEG can objectively capture short-term neurophysiological changes associated with sensory perception. Supportive dermatological evaluations are included to assess whether the direction of EEG changes aligns with visible or perceived skin responses, serving as contextual evidence rather than primary clinical endpoints. Validating EEG in this context may support its future application as a standardized tool for capturing skin-brain interactions and establishing objective neurosensory markers in dermatology research.

## Materials and methods

Ethical conduct of the study 

This exploratory clinical investigation was conducted in accordance with the ethical principles outlined in the Declaration of Helsinki, the International Conference on Harmonization - Good Clinical Practice (ICH-GCP) guidelines, and the ethical requirements of the Indian Council of Medical Research (ICMR). All study procedures adhered to applicable national regulations governing human participant research.

The study protocol underwent independent ethical review and received approval from the ACEAS-Independent Ethics Committee (IEC) prior to study initiation (Approval Number: NB250043-NB-V, dated 04 November 2025). For transparency and public accessibility, the study was prospectively registered with the Clinical Trials Registry-India (CTRI) (Registration No.: REF/2025/11/116613) and ClinicalTrials.gov (NCT No.: NCT07333300).

Eligible subjects were enrolled only after providing written informed consent. The consent process detailed the study objectives, procedures, potential risks and benefits, and confidentiality safeguards, and emphasized the voluntary nature of participation, including the right to withdraw at any time without penalty. Appropriate remuneration for subjects’ time spent for evaluation and travel allowance was provided in accordance with IEC and institutional guidelines.

Study design

This exploratory, randomized, double-blind, controlled-arm clinical investigation was conducted at a single clinical center to evaluate the feasibility and validity of using a wearable EEG headband to assess neurosensory responses in adults with mild to moderate AD. The study was designed to minimize bias and ensure methodological rigor, with both investigators and subjects blinded to the assigned intervention.

Eligible subjects were randomly allocated in a 1:1 ratio to receive either the marketed topical product or the control (water). EEG assessments were conducted under controlled, standardized conditions to record cortical activity before and after product application. The EEG recordings focused on detecting short-term changes in α, β, and θ brainwave patterns associated with itch perception, sensory irritation, or soothing effects.

All procedures were completed during a single study visit, which included screening, baseline evaluations, product application, and serial post-application EEG recordings. The controlled clinical environment ensured uniformity in ambient conditions, product application technique, and timing of EEG acquisition.

As this was a validation study, all operational workflows and assessment procedures were standardized to ensure methodological reliability. The entire process, from participant handling to EEG acquisition and data recording, was carried out by trained and competency-validated clinical staff. Prior to study initiation, personnel underwent structured training on the study protocol, neurosensory evaluation techniques, headband operation, and data quality requirements. Process validation measures confirmed consistency, accuracy, and reproducibility across all study steps, strengthening the robustness and reliability of the EEG-derived outcomes.

The primary aim of this investigation was to validate the feasibility, sensitivity, and reliability of EEG metrics for neurosensory assessment in AD subjects, rather than to evaluate long-term product efficacy. Findings from this exploratory study are intended to support the development of future large-scale studies establishing EEG-based neurosensory markers in dermatological research.

Eligibility criteria

Adults aged 18 to 65 years with clinically confirmed mild to moderate AD were enrolled in the study. Disease severity was determined using the Eczema Area and Severity Index (EASI), with eligible subjects presenting moderate erythema and mild scratch marks as evaluated by a dermatologist. Enrolled participants were generally healthy males or non-pregnant, non-lactating females and were able to comply with study requirements, and provided written informed consent. Females of childbearing potential reported a negative pregnancy test and confirmed the use of reliable contraception throughout the study.

Subjects were excluded if they had other dermatological conditions, known cosmetic allergies, active skin infections, chronic illnesses, or recent use of systemic immunosuppressants, topical steroids, or moisturizers that could interfere with study assessments. Additional exclusion criteria included pregnancy or breastfeeding, substance abuse, participation in another clinical study within four weeks prior to enrolment, or any condition deemed unsuitable by the investigator.

Visits and evaluations procedures

The study consisted of a single in-clinic visit (Day 01), during which all study-related activities were completed. Upon arrival, subjects underwent screening procedures to confirm eligibility, followed by randomization and enrolment into their assigned study arm. Baseline assessments were conducted prior to product application, including evaluation of the AD-affected area and setup of the EEG headband.

**Figure 1:**
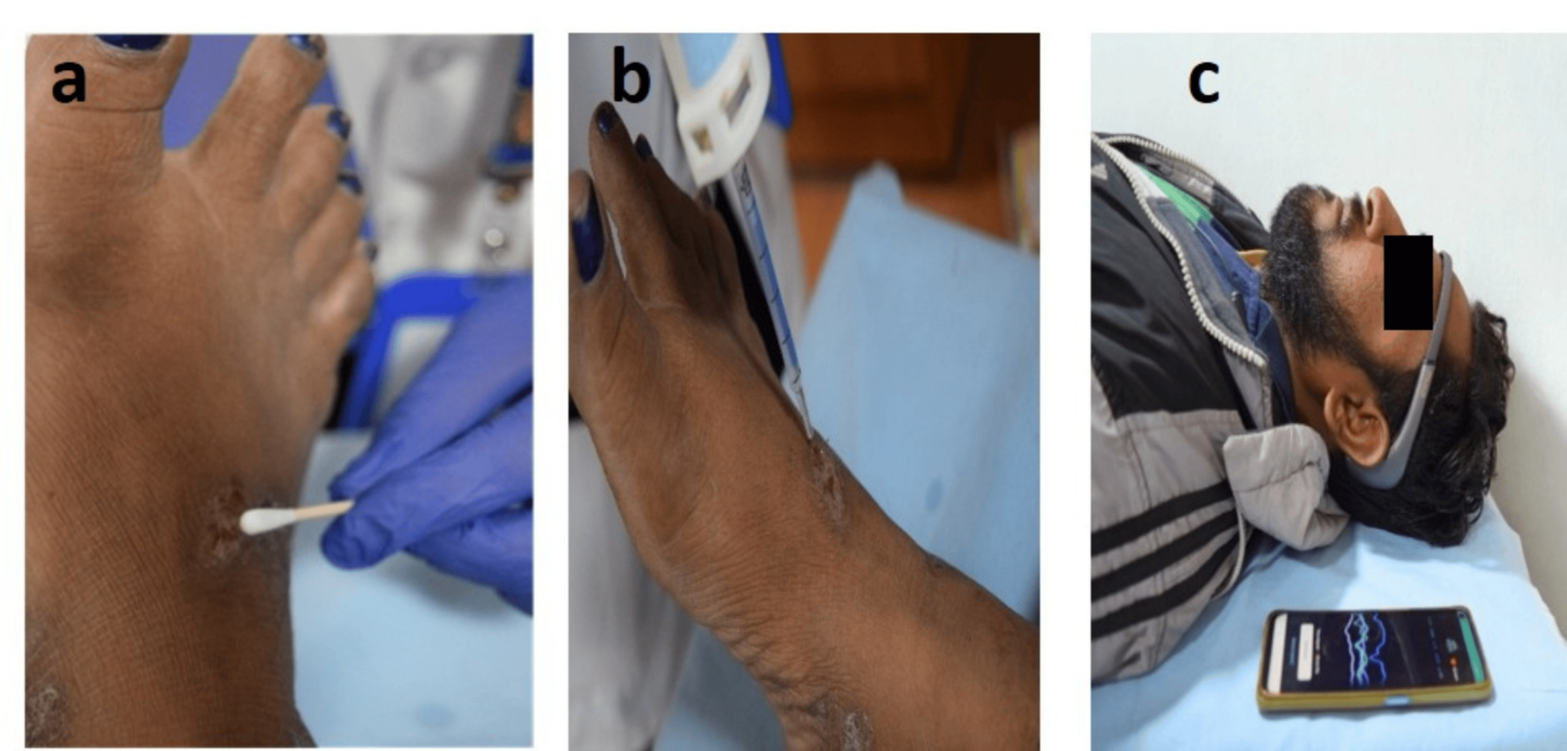
Study activity photographs illustrating key procedural steps: (A) 10% v/v lactic acid stimulation performed to induce a standardized sensory challenge, (B) application of the test product under controlled conditions, and (C) neurosensory assessment using the wearable EEG headband.

**Figure 2:**
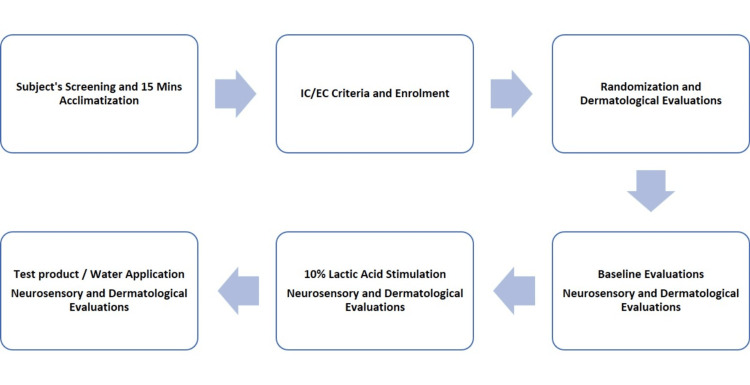
Study flow diagram illustrating participant screening, randomization, single-visit procedures, including baseline assessment, 10% v/v lactic acid stimulation, topical application, EEG evaluation, and completion of end-of-study assessments. IC: inclusion criteria, EC: exclusion criteria

Following baseline measurements, the assigned topical application - either the marketed product or control (water) - was administered under controlled conditions. Post-application assessments were performed at multiple time points for up to 30 minutes to monitor immediate neurosensory responses. EEG recordings served as the primary validation tool, capturing real-time brainwave activity. The analysis focused on changes in α, β, and θ wave patterns, which reflect cortical processing associated with sensory perception, comfort, and irritancy responses. Additionally, standardized digital photographs were captured using a Nikon D3300 camera to document any visible changes on the skin surface, if present. After completion of all assessments, end-of-study procedures were carried out, marking the conclusion of each participant’s involvement (Figures 1, 2).

Biosensing headband

Neurosensory assessment in this study was conducted using a wearable EEG headband (MUSE Biosensing headband; InteraXon Inc., Toronto, Canada), designed to record cortical electrical activity through dry electrodes positioned over the frontal and temporal scalp regions. The device operates on the fundamental principle of electroencephalography, detecting voltage fluctuations generated by synchronous neuronal activity in the cerebral cortex and recording these signals at the scalp surface. The acquired bioelectrical signals reflect underlying neural oscillations and are conventionally categorized into frequency bands, including α, β, delta (δ), θ, and gamma (γ), which are associated with different aspects of sensory processing, alertness, and neurosensory perception.

The EEG system incorporates integrated signal acquisition and filtering mechanisms to reduce noise and minimize artifacts related to motion and environmental interference. Its wearable configuration and dry-electrode design allow for standardized data acquisition in short, controlled clinical settings without the need for conductive gels or extensive setup. In the present study, EEG recordings were obtained at baseline and following topical application to quantify changes in oscillatory activity associated with neurosensory responses. The use of a wearable EEG system enabled consistent and reproducible measurement of neurosensory patterns, supporting its suitability for exploratory validation of EEG-based assessments in subjects with AD.

Randomization and blinding

Subjects were randomly assigned in a 1:1 ratio to receive either the marketed product or the control (water application). Randomization was performed using a pre-generated allocation sequence to ensure unbiased assignment. Both subjects and study personnel involved in product application, EEG assessments, and data analysis were blinded to treatment allocation throughout the study to minimize observational and assessment bias. The test product and control were identical in packaging, ensuring that neither the subjects nor the investigators could distinguish between them. Blinding was maintained until completion of data collection and locked analysis to preserve the integrity and objectivity of the study findings.

Study population

A total of 20 males and females aged between 18 to 65 years with mild to moderate AD were enrolled in this exploratory validation study. Subjects were randomized equally into two study arms, with 10 subjects assigned to the marketed product group and 10 subjects to the control (water) group. All subjects met the predefined eligibility criteria and demonstrated stable disease conditions suitable for controlled neurosensory assessment.

Sample size calculation

The sample size was estimated based on previously published data in patients with AD, where the mean ± standard deviation (SD) of the 4-Item Itch Questionnaire score was reported as 17.3 ± 2.5 in subjects with depressive symptoms and 13.1 ± 4.4 in those without depressive symptoms. From these values, a mean difference of 4.2 and a pooled SD of 3.58 were derived, yielding an effect size (Cohen’s d) of 1.17 [15].

A two-sample t-test was used for sample size estimation with a one-sided significance level of 0.05. Based on these assumptions, a sample size of approximately nine subjects per group was required to achieve 80% statistical power. To account for an anticipated dropout rate of approximately 10%, the planned enrolment was increased to 10 subjects per treatment arm.

## Results

Demographics and other baseline characteristics 

**Figure 3:**
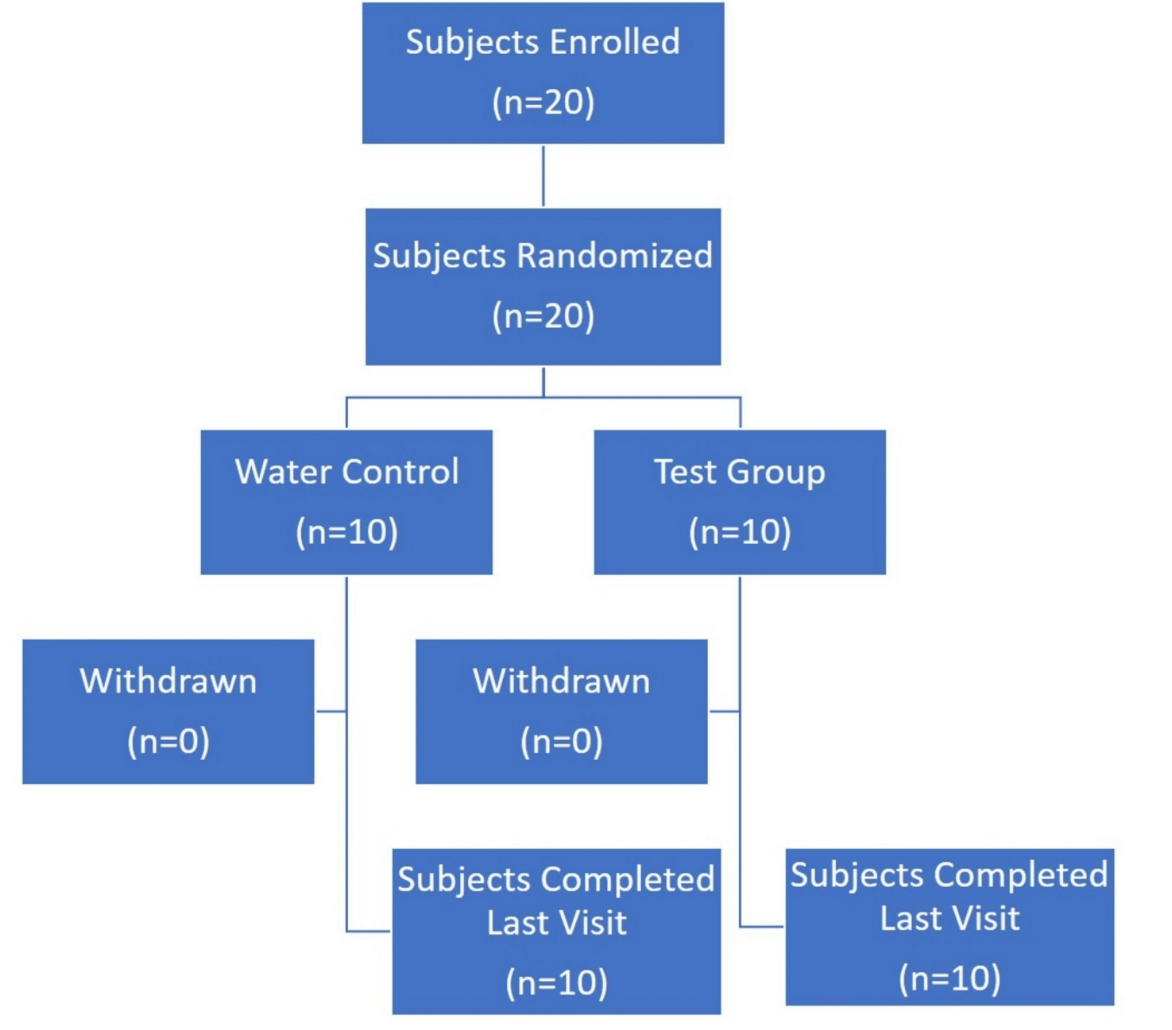
Subject Disposition Flowchart

The study comprised a total of 20 subjects, including seven females and 13 males. The mean age of the subjects was 41.50 years (Table 1, Figure 3).

**Table 1:** Demographic Representation

Demographic Details for Enrolled Subjects			
Parameter	Statistics	Total Enrolled Subjects, N= 20	Total Completed Subjects, N= 20
Gender M / F / TG	Female	7 (35%)	7 (35%)
	Male	13 (65%)	13 (65%)
Predominant Race	Asian	20 (100%)	20 (100%)
Medical History (Yes/No)	Yes	20 (100%)	20 (100%)
	No	0 (0%)	0 (0%)
Age (Years)	N	20	20
	Mean	41.50	41.50
	SD	8.27	8.27
	Median	40.50	40.50
	Min	19.00	19.00
	Max	55.00	55.00
Weight (kg)	N	20	20.00
	Mean	68.74	68.74
	SD	13.53	13.53
	Median	70.95	70.95
	Min	33.50	33.50
	Max	91.90	91.90
Height (cm)	N	20	20.00
	Mean	164.16	164.16
	SD	9.26	9.26
	Median	165.70	165.70
	Min	149.05	149.05
	Max	177.00	177.00

EEG evaluation and validation - test arm

At baseline, the EEG profile reflected a relatively calm and stable neurosensory state. The subject showed balanced low-frequency activity with moderate δ (1.7) and θ (1.1) waves, indicating a relaxed internal focus without signs of irritability or heightened sensory load. α activity (0.7) was present, suggesting a comfortable, calm-alert state typical of resting wakefulness. β (0.4) and γ (0.1) levels remained low, consistent with minimal cognitive arousal and absence of discomfort or sensory overstimulation. The heart rate of 90 bpm was within an expected physiological range for a resting condition. Overall, the baseline EEG demonstrates a steadily regulated cortical state, providing a neutral and stable reference point prior to 10% v/v lactic acid stimulation and product application.

During 10% v/v lactic acid stimulation, the EEG pattern showed a clear elevation across multiple frequency bands, reflecting an active cortical response to the induced sensory challenge. δ (2.0) and θ (1.6) activity increased noticeably, indicating enhanced somatosensory processing and greater internal awareness of the applied stimulus. α power (1.1) also rose, representing the brain’s attempt to regulate and adapt to the increased sensory input. Concurrently, modest increases in β (0.5) and γ (0.3) activity suggested heightened alertness and engagement of higher-order sensory integration mechanisms. The heart rate increased to 94 bpm, supporting a mild autonomic arousal response. Together, these changes indicate that 10% v/v lactic acid triggered a measurable but expected activation of sensory and regulatory pathways, demonstrating the subject’s cortical reactivity to a standardized irritant challenge.

**Figure 4:**
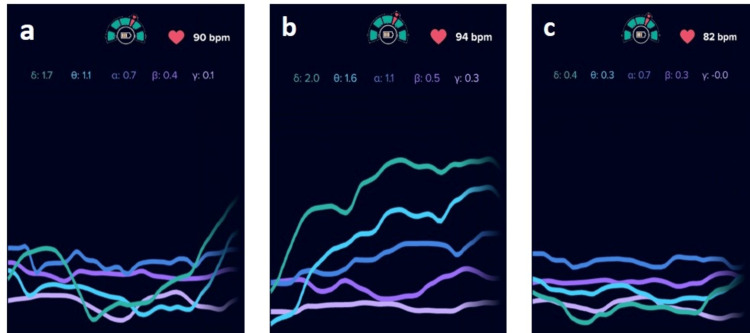
EEG evaluations in test product arm at different study phase - Subject #01 (a: baseline evaluations), (b: 10% v/v lactic acid stimulation) and (c: test product application) Electroencephalographic (EEG) evaluations in the test product arm for Subject #01 across different study phases: (a) baseline evaluation showing a stable and regulated cortical activity pattern, (b) 10% v/v lactic acid stimulation demonstrating increased EEG activity indicative of irritant-induced neurosensory activation, and (c) post–test product application showing normalization of EEG waveforms consistent with neurosensory calming and recovery.

Following application of the test product, the EEG profile demonstrated a return to a calm and well-regulated neurosensory state. δ (0.4) and θ (0.3) activity decreased substantially compared with the 10% v/v lactic acid phase, indicating a reduction in somatosensory load and diminished awareness of the prior irritant stimulus. α activity stabilized at 0.7, reflecting a comfortable calm-alert state consistent with relaxed wakefulness. β (0.3) and γ (~0.0) levels remained low, suggesting minimal arousal and an absence of irritation-related sensory integration. The heart rate decreased to 82 bpm, supporting a shift toward parasympathetic predominance. Together, these findings indicate that the test product effectively restored sensory balance and promoted neurosensory comfort following irritation, demonstrating good tolerability and a soothing influence on the skin-brain response pathway (Figure 4).

Dermatological evaluation - test arm

During dermatological assessment at baseline, the subject presented with mild to moderate irritation, dryness, erythema, itching, oedema, stinging, pruritus, and burning, consistent with the typical skin condition of an individual with AD. These clinical signs aligned with the baseline EEG pattern, which reflected a calm but sensitised neurosensory state. Upon 10% v/v lactic acid stimulation, the dermatological findings shifted to moderate irritation, dryness, erythema, itching, oedema, stinging, pruritus, and burning, indicating a clear aggravation of cutaneous sensitivity, which corresponded with EEG changes showing increased cortical activation and heightened sensory processing. Following application of the test product, dermatological scores returned to normal values across all parameters, demonstrating effective soothing and recovery of the skin barrier. This improvement corresponded with the EEG results, which also returned to a stable, calm-alert profile with reduced sensory load.

Together, the dermatological outcomes and EEG findings show a consistent pattern, confirming that both objective neurosensory measures and clinical skin evaluations follow the same trajectory across baseline, irritation, and recovery phases.

EEG evaluation and validation - control arm 

At baseline, the EEG profile indicated a well-regulated and relaxed neurosensory state. δ (0.1) and θ (0.3) levels were low, reflecting minimal somatosensory load and an absence of internalized discomfort or irritation signals. α activity (0.6) was present and stable, consistent with a calm-alert state typical of comfortable resting wakefulness. β (0.6) remained within normal physiological range, indicating balanced cognitive and sensory processing without signs of stress or hyperarousal. γ activity (0.1) was minimal, suggesting no evidence of burning, stinging, or heightened sensory integration. Heart rate was low at 60 bpm, supporting a parasympathetic-dominant resting condition.

Following 10% v/v lactic acid stimulation, the EEG pattern showed a noticeable increase in cortical activity compared with baseline, reflecting the skin’s response to the irritant challenge. δ activity rose to 0.9 and θ to 0.5, indicating enhanced somatosensory engagement and internal processing of the applied stimulus. α levels remained stable at 0.6, suggesting that although sensory input increased, the subject maintained a moderate calm-alert regulatory state. β activity (0.4) showed a mild rise, representing a natural increase in attentional and sensory monitoring as the skin reacted to the stimulus. γ activity stayed low (−0.1), indicating that the perceived stimulus did not provoke significant nociceptive or burning-type cortical integration.

**Figure 5:**
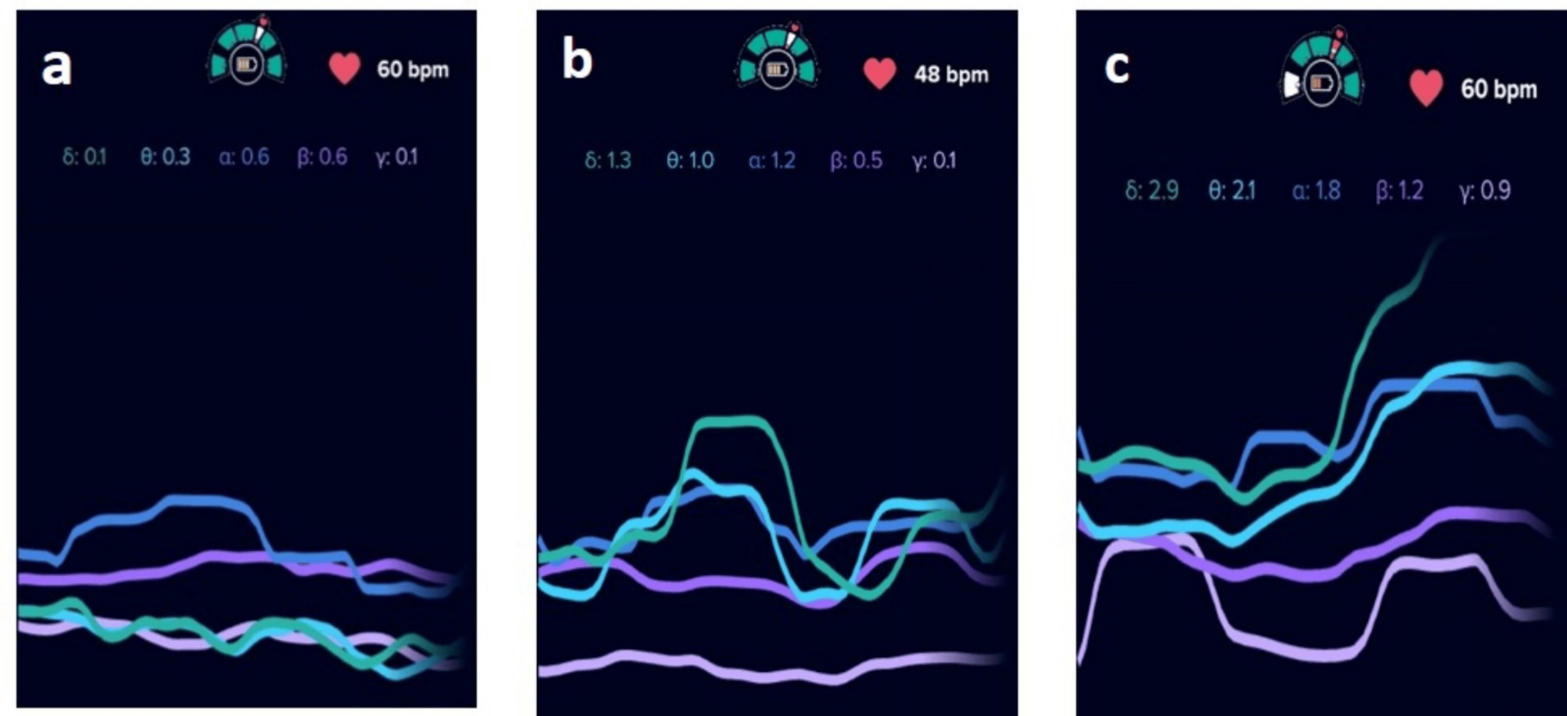
EEG evaluations in test product arm at different study phase (a: baseline evaluations), (b: 10% v/v lactic acid stimulation) and (c: water application). Electroencephalographic (EEG) evaluations in the test product arm across different study phases: (a) baseline evaluation showing a stable and regulated cortical activity pattern, (b) 10% v/v lactic acid stimulation demonstrating increased EEG activity indicative of irritant-induced neurosensory activation, and (c) water application showing persistent or variable EEG activity without clear neurosensory normalization.

Following water application, the EEG profile showed a general increase in activity across all frequency bands compared with baseline and 10% v/v lactic acid conditions, though without a clear soothing or stabilizing effect. δ (2.9) and θ (2.1) values rose substantially, indicating enhanced somatosensory processing and internal awareness, suggesting that the skin remained reactive rather than calmed. α activity increased to 1.8, reflecting cortical adjustment to ongoing sensory input rather than relaxation. β (1.2) and γ (0.9) elevations further indicated heightened alertness and active sensory integration, implying that low-level irritation or awareness may have persisted even with water alone. Despite these increases, the heart rate remained low at 60 bpm, showing no significant autonomic stress response (Figure 5).

Dermatological evaluation - control arm

During dermatological assessment at baseline, the subject presented with mild to moderate irritation, dryness, erythema, itching, oedema, stinging, pruritus, and burning, consistent with the expected clinical picture of AD. These findings aligned with the calm but sensitised baseline EEG pattern. Upon 10% v/v lactic acid stimulation, the dermatological scores increased to moderate levels across all parameters, reflecting heightened cutaneous reactivity. This escalation corresponded with the EEG findings, which showed increased δ, θ, β, and γ activity, indicating enhanced sensory processing and irritant response. Following water (control) application, the dermatological assessments remained moderate for irritation, dryness, erythema, itching, oedema, stinging, pruritus, and burning, demonstrating that simple hydration did not meaningfully reduce skin reactivity.

The EEG results mirrored this outcome, showing elevated activity across frequency bands and an absence of neurosensory calming, confirming that both dermatological and EEG evaluations followed the same pattern - baseline mild-moderate, 10% v/v lactic acid moderate, and water application maintaining moderate reactivity without improvement.

**Figure 6:**
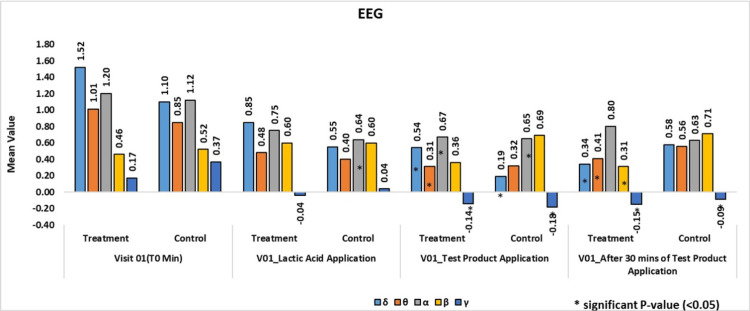
Change in the wavelengths before application, lactic acid stimulation and after application of the test product in treatment and control group. Changes in EEG wavelength activity before application (baseline), following lactic acid stimulation, and after application of the test product or placebo in the treatment and control groups, illustrating neurosensory responses across irritation and recovery phases.

The statistical analysis presented in the tables summarizes the comparative evaluation of EEG frequency band values, including δ, θ, α, β, and γ wavelengths, across the assessed study phases. The corresponding p-values indicate that the observed differences were statistically non-significant, suggesting the absence of meaningful inferential differences between the compared conditions. These findings are consistent with the exploratory and validation-focused nature of the study, where the primary objective was to assess feasibility, sensitivity, and directional neurosensory patterns rather than to establish statistically significant group differences. The results therefore support descriptive interpretation of EEG wavelength behavior in relation to neurosensory evaluation (Tables 2-6, Figure 6).

**Table 2:** Descriptive statistics of the δ wavelength Descriptive statistical summary of delta (δ) EEG wave activity in the test product and control arms at baseline and post-intervention time points during the single study visit. Values are presented as mean, SD, median, minimum, and maximum. P-values indicate within-group comparisons relative to baseline, demonstrating directional changes in neurosensory activity over time

Arm	Statistics	Visit 01(T0 Min)	Visit 01_Lactic Acid Application (T5 Min)	Visit 01(T15 Min) Test Product Application	Visit 01(T50 Min) After 30 mins of Test Product Application
		δ			
Treatment (N=10)	Mean	1.52	0.85	0.54	0.34
	SD	0.86	0.82	0.87	0.60
	Median	1.55	0.55	0.25	0.10
	Min	0.40	-0.10	-0.40	-0.30
	Max	2.80	2.10	2.10	1.20
	Test statistics (t)	-	-2.14	-3.74	-3.11
	P-value	-	0.06149	0.00466	0.01257
Control (N=10)	Mean	1.10	0.55	0.19	0.58
	SD	0.87	0.58	0.46	1.01
	Median	0.85	0.50	0.20	0.15
	Min	0.10	-0.10	-0.40	-0.10
	Max	2.90	1.90	0.90	2.90
	Test statistics (t)	-	-1.75	-2.66	-1.29
	P-value	-	0.11467	0.02613	0.22997

**Table 3:** Descriptive statistics of the θ wavelength Descriptive statistical summary of theta (θ) EEG wave activity in the test product and control arms at baseline and post-intervention time points during the single study visit. Values are presented as mean, SD, median, minimum, and maximum. P-values indicate within-group comparisons relative to baseline, demonstrating directional changes in neurosensory activity over time.

Arm	Statistics	Visit 01(T0 Min)	Visit 01_Lactic Acid Application (T5 Min)	Visit 01(T15 Min) Test Product Application	Visit 01(T50 Min) After 30 mins of Test Product Application
		θ			
Treatment (N=10)	Mean	1.01	0.48	0.31	0.41
	SD	0.65	0.54	0.41	0.29
	Median	0.85	0.35	0.15	0.40
	Min	0.00	0.00	0.00	0.00
	Max	2.00	1.60	1.10	0.80
	Test statistics (t)	-	-2.09	-3.43	-2.40
	P-value	-	0.06644	0.00757	0.03983
Control (N=10)	Mean	0.85	0.40	0.32	0.56
	SD	0.79	0.41	0.35	0.66
	Median	0.50	0.25	0.30	0.30
	Min	0.20	-0.10	-0.20	0.10
	Max	2.60	1.20	1.00	2.10
	Test statistics (t)	-	-1.71	-1.76	-1
	P-value	-	0.12123	0.11187	0.34464

**Table 4:** Descriptive statistics of the α wavelength Descriptive statistical summary of alpha (α) EEG wave activity in the test product and control arms at baseline and post-intervention time points during the single study visit. Values are presented as mean, SD, median, minimum, and maximum. P-values indicate within-group comparisons relative to baseline, demonstrating directional changes in neurosensory activity over time.

Arm	Statistics	Visit 01(T0 Min)	Visit 01_Lactic Acid Application (T5 Min)	Visit 01(T15 Min) Test Product Application	Visit 01(T50 Min) After 30 mins of Test Product Application
		α			
Treatment (N=10)	Mean	1.2	0.75	0.67	0.8
	SD	0.69	0.21	0.21	0.25
	Median	1.1	0.7	0.65	0.85
	Min	0.4	0.5	0.3	0.2
	Max	2.7	1.1	1.1	1.1
	Test statistics (t)	-	-2	-2.51	-1.71
	P-value	-	0.07625	0.03345	0.12194
Control (N=10)	Mean	1.12	0.64	0.65	0.63
	SD	0.64	0.23	0.3	0.49
	Median	0.95	0.7	0.65	0.45
	Min	0.6	0.3	0.2	0.2
	Max	2.6	1	1.1	1.8
	Test statistics (t)	-	-2.4	-2.38	-1.71
	P-value	-	0.03981	0.04148	0.12185

**Table 5:** Descriptive statistics of the β wavelength Descriptive statistical summary of beta (β) EEG wave activity in the test product and control arms at baseline and post-intervention time points during the single study visit. Values are presented as mean, SD, median, minimum, and maximum. P-values indicate within-group comparisons relative to baseline, demonstrating directional changes in neurosensory activity over time.

Arm	Statistics	Visit 01(T0 Min)	Visit 01_Lactic Acid Application (T5 Min)	Visit 01(T15 Min) Test Product Application	Visit 01(T50 Min) After 30 mins of Test Product Application
		β			
Treatment (N=10)	Mean	0.46	0.60	0.36	0.31
	SD	0.21	0.49	0.16	0.12
	Median	0.40	0.50	0.35	0.30
	Min	0.20	0.10	0.10	0.10
	Max	0.80	1.70	0.70	0.50
	Test statistics (t)	-	0.91	-1.27	-3.31
	P-value	-	0.38801	0.23671	0.00911
Control (N=10)	Mean	0.52	0.60	0.69	0.71
	SD	0.31	0.53	0.52	0.65
	Median	0.55	0.50	0.55	0.45
	Min	0.00	0.10	0.10	0.10
	Max	1.00	1.90	1.90	1.80
	Test statistics (t)	-	0.35	0.78	0.71
	P-value	-	0.73092	0.45366	0.49711

**Table 6:** Descriptive statistics of the γ wavelength Descriptive statistical summary of gamma (γ) EEG wave activity in the test product and control arms at baseline and post-intervention time points during the single study visit. Values are presented as mean, SD, median, minimum, and maximum. P-values indicate within-group comparisons relative to baseline, demonstrating directional changes in neurosensory activity over time.

Arm	Statistics	Visit 01(T0 Min)	Visit 01_Lactic Acid Application (T5 Min)	Visit 01(T15 Min) Test Product Application	Visit 01(T50 Min) After 30 mins of Test Product Application
		γ			
Treatment (N=10)	Mean	0.17	-0.04	-0.14	-0.15
	SD	0.34	0.24	0.29	0.29
	Median	0.05	-0.15	-0.15	-0.20
	Min	-0.30	-0.30	-0.60	-0.60
	Max	0.90	0.30	0.40	0.20
	Test statistics (t)	-	-1.55	-2.51	-2.43
	P-value	-	0.15521	0.03312	0.03775
Control (N=10)	Mean	0.37	0.04	-0.18	-0.09
	SD	0.47	0.38	0.14	0.41
	Median	0.20	-0.05	-0.15	-0.20
	Min	-0.20	-0.40	-0.40	-0.40
	Max	1.30	0.80	0.00	0.90
	Test statistics (t)	-	-1.59	-3.68	-2.55
	P-value	-	0.14649	0.00504	0.03109

Comparative summary

Overall, EEG recordings demonstrated clear differentiation between the test product and control arms. Both groups exhibited increased cortical activity following 10% v/v lactic acid stimulation, confirming EEG sensitivity to irritant-induced neurosensory activation. However, only the test product arm showed normalization of EEG activity following application, whereas the control arm exhibited sustained or increased activity across frequency bands. These findings support the ability of EEG to distinguish between soothing and non-soothing topical interventions.

EEG-dermatological concordance

Across baseline, irritant challenge, and post-application phases, EEG findings followed the same directional trends as dermatological evaluations in both study arms, indicating concordance between objective neurosensory measures and clinical skin observations.

The reproducibility of EEG responses across baseline and irritant phases, along with differential post-application patterns between test and control arms, confirms the feasibility and sensitivity of the wearable EEG headband for neurosensory evaluation in AD.

## Discussion

This exploratory clinical investigation demonstrates that a wearable EEG headband can reliably capture neurosensory responses associated with baseline skin status, irritant-induced activation, and post-application recovery in adults with mild to moderate AD. The observed EEG changes across frequency bands support the feasibility and sensitivity of EEG as an objective neurosensory assessment tool in dermatological research, addressing a key limitation of reliance on subjective symptom reporting alone.

At baseline, EEG profiles across both study arms reflected a calm and regulated cortical state, characterized by stable α activity and low β and γ power, consistent with resting wakefulness and minimal sensory distress. Such patterns have been previously associated with balanced sensory processing and low central nervous system arousal, even in individuals with chronic inflammatory skin conditions. These findings confirm the suitability of baseline EEG recordings as a neutral reference point for subsequent neurosensory comparisons.

Lactic acid (10% v/v) stimulation produced a reproducible increase in δ, θ, β, and γ activity across both test and control arms, reflecting heightened somatosensory processing and cortical engagement. Prior neurophysiological studies have demonstrated that itch and chemical irritants activate cortical networks involved in sensory discrimination, internal awareness, and affective processing, often manifested as increased low- and mid-frequency EEG oscillations. The consistent EEG response to 10% v/v lactic acid observed in this study validates EEG sensitivity to standardized irritant challenges in AD populations.

Following application of the test product, EEG activity shifted toward normalization, with reductions in δ and θ activity and stabilization of α rhythms, indicating attenuation of sensory load and restoration of neurosensory balance. Reduced β and γ activity further suggested diminished irritant-driven cortical integration. Similar EEG changes have been associated with relief from sensory discomfort and improved neurosensory regulation in prior experimental paradigms. In contrast, control (water) application failed to produce comparable EEG normalization, with sustained or increased activity across frequency bands, indicating persistent sensory awareness rather than neurosensory calming.

Dermatological evaluations were included as a supportive measure to contextualize EEG findings. Across all study phases, dermatological assessments followed the same directional pattern as EEG results - baseline mild-to-moderate symptoms, worsening after irritant exposure, and improvement only in the test product arm. Previous research suggests that central processing of itch and irritation closely parallels clinical skin manifestations, reinforcing the relevance of EEG as a marker of skin-brain interaction [16]. While dermatological outcomes were not primary endpoints, their alignment with EEG trends strengthens confidence in the physiological validity of the EEG-derived neurosensory measures.

Recent advancements in low-cost, portable EEG systems, such as the headband, have substantially expanded opportunities for conducting neurosensory research in clinical and real-world settings. Improvements in signal acquisition quality and artifact management now allow reliable detection of meaningful cortical activity using brief, time-efficient assessment protocols. Previous studies have demonstrated that wearable EEG devices are capable of quantifying established event-related potential (ERP) components, including N200, P300, and reward positivity, within short experimental paradigms. In the present study, the successful capture of distinct EEG changes across baseline, irritant stimulation, and post-application phases further supports the capability of portable EEG technology to detect functionally relevant neurosensory responses within a single, short-duration clinical visit. These findings reinforce the feasibility of integrating wearable EEG systems into dermatological research where rapid, objective, and participant-friendly neurosensory assessments are required.

Overall, these findings support the use of wearable EEG technology as a feasible and objective methodology for neurosensory evaluation in dermatology. The ability of EEG to distinguish between irritant-induced activation, product-induced soothing, and control response highlights its potential utility in early-phase product testing, mechanistic skin-brain research, and development of standardized neurosensory endpoints.

This study has limitations, including its exploratory nature, small sample size, and single-visit design, which limit generalizability. Future studies should incorporate larger cohorts, longitudinal follow-up, and quantitative symptom scales to further establish EEG reproducibility and clinical relevance. Nonetheless, the present investigation successfully validates EEG as a sensitive and reliable tool for neurosensory assessment in AD, with supportive dermatological correlation reinforcing its interpretability.

## Conclusions

This exploratory validation study demonstrates that a wearable EEG headband is a feasible, reliable, and sensitive tool for assessing neurosensory responses in adults with mild to moderate AD. EEG recordings consistently captured cortical changes associated with baseline conditions, irritant-induced activation, and post-application recovery, confirming its ability to objectively detect short-term neurosensory modulation. The differentiation between soothing effects following test product application and the absence of neurosensory normalization after control further supports the discriminatory capacity of EEG in dermatological research settings.

Supportive dermatological evaluations followed the same directional pattern as EEG findings, reinforcing the physiological relevance of EEG-derived measures without serving as primary clinical endpoints. Together, these results validate EEG as an objective methodology for investigating skin-brain interactions and provide a foundation for its application in future dermatology studies. Larger, longitudinal investigations are warranted to further establish reproducibility, refine neurosensory endpoints, and integrate EEG-based measures into standardized clinical research frameworks. 
